# News and Notes

**Published:** 1902-04

**Authors:** 


### *NEWS AND NOTES.*

(We are indebted to the *Medical Age* for many of these notes.)

---

THE *Atlanta Medical and Surgical Journal* and *Southern Medical Record* having been consolidated into the ATLANTA JOURNAL-RECORD OF MEDICINE, exchanges will please be sent only to the latter. We are getting many duplicates.

---

VIRCHOW is progressing slowly toward recovery.

---

THE second national Italian Antipellagra Congress will meet at Bologna next May.

---

THE *Cleveland Medical Journal* has been incorporated with a capital stock of $6,000.

---

A SOLARIUM is being built at the Government Sanatorium for tuberculosis at Fort Bayard, New Mexico.

---

DR. W. W. BURNS, of Polo, Ill., who is now in his eighty-first year, has been in active practice for fifty-four years.

---

MORE than 50,000 francs have been donated for the erection of the monument to Ollier, the surgeon, of Lyons, France.

---

ANDREW CARNEGIE has given $50,000 for the erection of a hospital in Pittsburg to give first aid to injured workmen.

---

DR. HENRY CARSON, of Forest City, Pa., is dead at the age of 108. He continued to practice his profession up to last October.

THE Battle Creek Sanitarium which was recently burned, entailing a loss estimated at $400,000, will be rebuilt immediately.

---

AT the public exercises held by the University of Pennsylvania on Washington's birthday, Dr. S. Weir Mitchell read an original poem.

---

THE important post of Medical Director of the St. Louis World's Fair has been filled by the appointment of Dr. Leonidas H. Laidley.

---

DR. MATTHEW D. MANN, who is also one of the park commissioners, has been elected President of the Society for Beautifying Buffalo.

---

THE authorities of Yale College have announced that hereafter the course in the Yale Medical School can be made in three years instead of four.

---

THE latest from the New York Hospital Department is that diphtheritic infection may linger from 12 days to 40 days about the patient after convalescence is established.

---

HINDOO twins, similar to the famous Siamese twins, were recently separated by means of a surgical operation performed by Dr. Doyen, of Paris. One of them has since died.

---

ONE of the measures adopted in the campaign against charlatanry being waged in Germany is the publication in the medical journals of descriptions of the nature of the most widely advertised quack medicines.—*Exchange.*

---

THE second meeting of the Congress of Russian Surgeons was held at Moscow in the early part of January under the presidency of Prof. W. Rasumowski, of Kasan. The congress was attended by about 200 members.

---

SURGEON JOSEPH J. KINYOUN, U. S. Marine Hospital Service, stationed at Detroit since his transfer from San Francisco a year ago, has resigned and will reside in Philadelphia and devote himself to bacteriologic work.

---

A HOSPITAL car was attached to each of the five trains required to transport the 29th Infantry from Fort Sheridan to San Francisco, *en route* to the Philippines. This was rendered necessary by the prevalence of measles at the post.

---

RECOMMENDATION has been made by Surgeon-General Rixey of the navy that the medical corps of the navy be increased by forty medical officers, this increase to be made by the addition of fifteen surgeons and twenty-five assistant surgeons.

---

THE Medical Department of McGill University has received a large sum of money from M. E. S. Bronson, of Ottowa, for research-work in possible methods for the cure of consumption. These investigations will be undertaken by Dr. A. G. Nichol, under the supervision of Prof. J. G. Adami.

---

GEORGIA MEDICAL SOCIETY, OF SAVANNAH.—The following officers were elected: President, Dr. John a Crowther; Vice-President, Dr. A. A. Morrison; Treasurer, Dr. Charles B. Lanneau; Recording Secretary, Dr. J. O. Cook; Corresponding Secretary, Dr. H. H. Martin; Librarian, Dr. J. G. Van Marter.

---

DR. COLOMAN MULLER, Professor of Medicine in the University of Hungary and physician in chief to the Rochus Hospital, has been raised to the dignity of a seat in the Hungarian House of Magnates. Dr. Muller was organizing secretary of the Eighth Congress of Hygiene and Dermography, which was held at Budapest in 1894.

---

PRESIDENT ELIOT, of Harvard University, recently announced to the medical faculty that Mr. John D. Rockefeller proposed to give $1,000,000, in aid of the medical school, provided other friends of the University would raise a sum of money in the neighborhood of $500,000, to be used by the medical school for land, buildings, or endowments.

---

A DENTAL practice bill has been introduced in the New York State legislature providing that no degree in dentistry shall be conferred in that State unless the candidate has satisfactorily completed a three-year course in an institution registered by the regents, or has had five years' actual practice in operative and mechanical dentistry subsequent to the registration of his certificate as a student of the Board of Examiners.

---

SURGEON-GENERAL STERNBERG has recommended that the Contract-Surgeon James E. Mead have a commission as captain and assistant surgeon of volunteers on account of gallant conduct and professional zeal in the Philippines. This action is taken on the recommendation of First Lieutenant Louis Van Schaick, Fourth Infantry, indorsed by General Chaffee and Lieut.-Col. B. F. Pope, chief surgeon of the Division of the Philippines.

---

DR. CLINTON CUSHING, of San Francisco, has been sued by Mary Stephens, a daughter of Thomas Fauber, of Yountville, Cal., for $50,000 damages. It is alleged that in February, 1896, at the Lane Hospital, in San Francisco, the defendant performed a surgical operation and left a drainage tube four or five inches long in her body. This drainage tube, it is alleged, was afterwards removed by an operation at the California Woman's Hospital, October, 1900.

---

THE Victoria Cross, "For valor," has been conferred upon Surgeon-Captain T. J. Crean, of the First Imperial Light Horse. The following is the official statement of the cause: During the action with De Wet at Tygerskloof, on December 18, 1901, this officer continued to attend to the wounded in the firing line, under a heavy fire at only 150 yards range, after he had himself been wounded, and only desisted when he was hit a second time, and, as it was at first thought, mortally wounded.—*Exchange.*

---

THE War Department has recently directed that one of the seacoast defenses near Baltimore, Md., shall hereafter be known as Battery Lazear. According to the order this action was taken in honor of Dr. Jesse W. Lazear, late an acting assistant surgeon, United States Army, who, while on a visit to Las Animas Hospital, Havana, Cuba, September 13, 1900, and while collecting blood from yellow-fever patients for scientific study, was bitten by a Culex mosquito and deliberately allowed it to satisfy its hunger, and as a result contracted yellow fever, of which he died September 25, 1900, thus by his self-sacrifice positively determining that the mosquito carries yellow-fever infection.

---

IN the last dozen years influenza has become not only an important factor in the mortality of the city of Chicago, says the *Journal of the American Medical Association*, but as a mischievous complication has notably increased the death-rate from pneumonia, the deaths from which have increased fifty per cent. since influenza appeared in 1889. Cancer is increasing with frightful rapidity. In the decade ended in 1870 there were only 7.3 cancer deaths in every 1,000 deaths from all causes in Chicago. In the decade ended in 1900 there were 28.9 such deaths in every 1,000, an increase in thirty years of more than 80 per cent. Last year there were 1,003 deaths from this disease in a total of 24,406 deaths from all causes—a proportion of more than 41 in every 1,000, instead of 7.3 in the decade of 1870, of the 14.5 in that of 1880, of the 19.8 in that of 1890, and of the 28.9 in that of 1900.

---

WE have known buboes to be called "pigs," and that appellation is rather suggestive of a rich imagination, but we must admit that the French Tanners take the lead in bestowing on disease names that smack decidedly of tenderness. According to Brocq and Landry (*Annales de Dermatologie et de Syphiligraphie*), a form of "occupational" ulcer, chiefly affecting the fingers, is frequently met with among the dyers of leather, attributed to the penetration of caustic fluids into the fissures of the skin. The ulcers are described as extremely painful, as round as if cut out with a punch, surrounded with a reddish infiltrated wall, and often accompanied by surrounding redness and infiltration. The pain is so great that the workmen present themselves early for treatment; nevertheless, the healing of these ulcers is apt to linger. The disease must be a sore infliction; however, it is called by such endearing names as *pigeonneau* (the young pigeon), *rossignol* (the nightingale), *tourtereau* (the young turtle-dove). Perhaps it is possible to mitigate one's sufferings by petting the lesions that give rise to them!—*New York Medical Journal.*

---

HELEN KELLER'S FIRST EARNINGS.—There is a pretty story in connection with the series of articles which Hellen Keller, the wonderful blind girl, has written for *The Ladies' Home Journal*, telling about her own life from infancy to the present day. She always has shrunk from the publicity which follows successful literary work, and it was with great difficulty that she was persuaded to take up the task of preparing her autobiography. She had, however, set her heart on owning an island in Halifax harbor for a summer home, and in a spirit of fun the editor of *The Journal* offered to buy it for her, or to provide the means to buy it. When the work of writing appeared especially irksome Miss Keller was reminded of her desire to become a land-holder, and it spurred her on. Just before Christmas she completed the first chapter of her marvelous story; and on Christmas morning she received from her publishers a check for a good round sum. Her delight may be imagined, for this was the first money of any account which she had ever earned. "It is a fairy tale come true," she said. Whether she will really carry out her plan to buy the island remains to be seen.

---

THE NATHAN LEWIS HATFIELD PRIZE FOR ORIGINAL RESEARCH IN MEDICINE.—The College of Physicians of Philadelphia announces through its committee that the sum of five hundred dollars will be awarded to the author of the best essay in competition for the above prize. Subject: "The Relation between Chronic Suppurative Processes and Forms of Anaemia." Essays must be submitted on or before March first, 1903. Each essay must be typewritten, designated by a motto or device, and accompanied by a sealed envelope bearing the same motto or device and containing the name and address of the author. No envelope will be opened except that which accompanies the successful essay. The committee will return the unsuccessful essays if reclaimed by their respective writers or their agents within one year. The committee reserve the right not to make an award if no essay is considered worthy of the prize. The treatment of the subject must, in accordance with the conditions of the trust, embody original observations or researches or original deductions. The competition shall be open to members of the medical profession and men of science in the United States. The original of the successful essay shall become the property of the College of Physicians. The Trustees shall have full control of the publication of the memorial essay. It shall be published in the Transactions of the College, and also when expedient as a separate issue. Address J. C. Wilson, M.D., Chairman, College of Physicians, 219 South Thirteenth Street, Philadelphia, Pa.

---

THE American Association of Urologists was organized on February 22, 1902, essentially for the purpose of further development of the study of the urinary organs and their diseases. Although most of the founders of the Association are specialists in genito-urinary diseases, membership is not limited to those engaged exclusively in this specialty. Thus gynecologists who embrace renal and vesical surgery in their work, are among the founders, as are several gentlemen who devote themselves to the microscopy and chemistry of the urine, as well as a number of practitioners interested in the study of the kidney from a medical standpoint. The Association consists of active, corresponding and honorary members, and is in great measure modelled upon the plan of the Societé Francaise d'Urologie, modified to suit American circumstances and conditions. Whenever possible, the branch associations throughout the United States, British Possessions and Spanish America, will hold their meetings on the same evenings as does the parent association in New York, (the first Wednesday in each month). The work of the Association is principally clinical, for the demonstration of new methods of the technique of examination and treatment. The annual meeting of the American Association of Urologists will be held on the last day and the day following the annual meeting of the American Medical Association. The officers of the Association are: Ramon Guiteras, M.D., President; Wm. K. Otis, M.D., Vice-President; John Van der Poel, M.D., Treasurer; Ferd. C. Valentine, Secretary; A. D. Mabie, M.D., Assistant Secretary.

THE following is the programme of the fifty-third annual session of the Medical Association of Georgia, to be held in Savannah, April 16, 17 and 18, 1902:

1. The Medical Aspect of Life Insurance. L. Amster, M.D., Atlanta.

2. A Symposium on the Pathology and the Treatment of Typhoid Fever, with Special Reference to the so-called Eliminative, or Woodbridge Treatment. James B. Baird, M.D., Atlanta.

3. Accidental Cures, Monstrosities and Adventitious Growths. F. M. Brantly, M.D., Senoia.

4. Vesico-Vaginal Fistula. W. Jay Bell, M.D., Atlanta.

5. Tuberculosis. George Brown, M.D., Atlanta.

6. Caustic Potash—Concentrated Lye—A Dangerous Commodity. W. L. Bullard, M.D., Columbus.

7. The Necessity of Alterative in Doubtful Cases. J. Lawton Hiers, M.D., Savannah.

8. New Remedies in the Treatment of Eye Diseases. A. W. Calhoun, M.D., Atlanta.

9. Surgery of the Gall Bladder with Report of two cases. Hunter P. Cooper, M.D., Atlanta.

10. X-Ray Apparatus and X-Ray Methods and the Lines along which Progress has been made. Eugene R. Corson, M.D., Savannah.

11. Trachoma. J. M. Crawford, M.D., Atlanta.

12. Treatment of Chronic Gastritis. J. D. Cromer, M.D., Atlanta.

13. The X-Ray Treatment of Cancers. Jno. W. Daniel, M.D., Savannah.

14. Ectopic Pregnancy. E. C. Davis, M.D., Atlanta.

15. A Splenectomy with Remarks. John G. Earnest, M.D., Atlanta.

16. Use of Spermine in Treatment of Nervous Diseases. Eugene B. Elder, M.D., Indian Springs.

17. Malarial Neuritis. St. J. B. Graham, M.D., Savannah.

18. The Human and Business Side of the Medical Profession. W. B. Hardman, M.D., Harmony Grove.

19. The Treatment of Uterine Fibroids. Virgil O. Hardon, M.D., Atlanta.

20. Continued Malarial Fever, Erroneously Termed "Slow Fever." V. O. Harvard, M.D., Arabi.

21. Clinical Experience with Adrenalin. Arthur G. Hobbs, M.D., Atlanta.

22. Flat Foot. Michael Hoke, M.D., Atlanta.

23. A Frequent, Serious and Important Complication of Typhoid Fever with Treatment. W. D. Hoyt, M.D., Rome.

24. Report of Some Cases of Injury to the Head and Spine. C. D. Hurt, M.D., Atlanta.

25. Gastric Hyperacidity—Its Pathology and Treatment. J. C. Johnson, M.D., Atlanta.

26. Report of Cases—Filaria Hominis Sanguinis with Pregnancy—Embolism following Labor—Transverse Laceration Perineum. R. R. Kime, M.D., Atlanta.

27. Neurasthenia—Its Classification and New Method of Treatment. J. Cheston King, M.D., Atlanta.

28. Tuberculosis. H. McHatton, M.D., Macon.

29. Personal Experiences in Operations upon the Carotid Arteries, consisting of 24 Ligations and 4 Excisions. W. P. Nicolson, M.D., Atlanta.

30. Nephrectomy in Tubercular and Calculous Pyelonephritis—An Artificial Vagina. Geo. H. Noble, M.D., Atlanta.

31. The Treatment of Typhoid Fever. C. T. Nolan, M.D., Marietta.

32. Kidney Diseases in the Insane—A Study of Six Hundred Urinalyses and Seventy Autopsies. M. L. Perry, M.D., Milledgeville.

33. Myometritis. Ernest H. Rawls, M.D., Savannah.

34. The Treatment of Catarrhal Deafness by Electrolysis of the Eustachian Tube. Dunbar Roy, M.D., Atlanta.

35. Some Headaches with Eye Strain. J. H. Shorter, M.D., Macon.

36. Report of Some Surgical Cases. W. Monroe Smith, M.D., Atlanta.

37. The Physician as a Social Economic Factor. E. J. Spratling, M.D., Forsyth.

38. Report Showing Clinics and Methods of Establishing Hearing in Deaf Mutes. M. M. Stapler, M.D., Macon.

39. Short Sight. Alex. W. Stirling, M.D., Atlanta.

40. The Diagnostic Importance of the Leucocytes in the Blood. J. M. Thomas, M.D., Griffin.

41. Variola. W. D. Travis, M.D., Covington.

42. Membranous Croup—Diphtheritic Laryngitis. S. A. Visanska, M.D., Atlanta.

43. Treatment of Ulcers. Willis F. Westmoreland, M.D., Atlanta.

44. Laminectomy—two cases. Howard J. Williams, M.D., Macon.

45. Preventive Medicine. W. P. Williams, M.D., Blackshear.

46. Syphilitic Dementia. James B. Dillard, M.D., Davisboro.

47. The Sin of the So-called Conservative Medical Treatment in Conditions Requiring Prompt Surgical Intervention. Floyd W. McRae, M.D., Atlanta.

48. Some Reasons why we should have a State Board of Health. E. C. Thrash, M.D., Neal.

49. The Pubescent and Adolescent Period, with a few Thoughts on Dress and Education. W. E. Fitch, M.D., Savannah.

50. Report of a case of Ectopic Pregnancy—Removal of Foetus at Full Term. W. S. Elkin, M.D., Atlanta.

51. A case of Diabetes Mellitus with Autopsy. Eugene E. Murphy, M.D., Augusta.

52. Some Habits of Malaria not Controlled by the Mosquito. Charles Hicks, M.D., Dublin.

